# An eye on long‐duration spaceflight: Controversies, countermeasures and challenges

**DOI:** 10.1113/EP091561

**Published:** 2025-07-03

**Authors:** Vincent Wing Sum Ng, Susan Patricia Mollan

**Affiliations:** ^1^ Birmingham Neuro‐Ophthalmology University Hospitals Birmingham NHS Foundation Trust Birmingham UK; ^2^ Department of Metabolism and Systems Science, School of Medical Sciences, College of Medical and Health University of Birmingham Birmingham UK

**Keywords:** astronaut, glucagon‐like peptide‐1 receptor agonists, microgravity, optic nerve, raised intracranial pressure, space flight‐associated neuroocular syndrome, vision

## Abstract

Space flight‐associated neuroocular syndrome (SANS) is a consequence of long‐duration space flight and is detected in two‐thirds of astronauts. In‐flight, this can cause a change in the refraction of the eyes, requiring graded hypermetropic ‘superfocus adjustable’ glasses, optic nerve head oedema and choroidal folds. While the optic disc oedema resolves on returning to gravitational force, the choroidal folds and axial length shortening remain. Controversy remains over the role of intracranial pressure in the development of these changes. A recent case report has re‐energised the debate as to whether nutraceuticals and an individual's genetic expression of the 1‐carbon metabolic pathway are a major component for the development of this condition. The strict 6° head down tilt bed rest platform remains the cornerstone for evaluating analogous pathological changes and trialling potential targeted therapies. The translational application of a glucagon‐like peptide 1 receptor agonist for the treatment of raised intracranial pressure in idiopathic intracranial hypertension may be of significance as a potential countermeasure for SANS. The aim of this narrative review is to explore the controversies in the integration of the presumed pathophysiological factors and to debate suitable countermeasures.

## Abstract (highlights)

- **What is the topic of this review?**
  This review focuses on spaceflight‐associated neuroocular syndrome (SANS), which is a consequence of long‐duration space flight and potentially poses a risk to astronaut eye health.
- **What advances does it highlight?**
  Three key factors are thought to influence the development of SANS: cephalic fluid shift secondary to microgravity; the effect of microgravity on ocular and intracranial venous outflow; and alterations in genetic expression of the 1‐carbon metabolic pathway. Head down tilt bed rest studies and terrestrial conditions, such as idiopathic intracranial hypertension, are current analogues providing surrogate evidence for the investigation and management of SANS.

## 1 INTRODUCTION

Space exploration presents a hostile environment to human physiology. During long‐duration space flights (LDSF) beyond low Earth orbit, astronauts encounter a range of neuro‐ocular changes. Space flight‐associated neuro‐ocular syndrome (SANS) is a pattern of eye changes that include optic disc oedema, choro‐retinal folds, globe flattening and change in refractive error (Mader et al., 2011). SANS may not be an exclusively orbital physiological response to LDSF, as post‐flight magnetic resonance imaging reveals a number of structural brain changes (Hupfeld et al., 2020; Kramer et al., 2015; 2020; Roberts et al., 2017; Van Ombergen et al., 2019). The pathophysiology of SANS is not clearly understood, although cephalic fluid shifts secondary to microgravity (μ‐gravity) and the potential effects of μ‐gravity on intracranial venous outflow are thought to be major environmental risk factors. Genetic polymorphisms in the 1‐carbon metabolic pathway have been associated with the development of SANS (Zwart et al., 2012). The pathophysiological changes are recorded by various non‐invasive methods, such as fundus photography, optical coherence tomography (OCT) imaging and magnetic resonance imaging used in terrestrial neuro‐ophthalmic clinical practice. Countermeasures for the sequelae of SANS have been considered with direct translation of treatments used in terrestrial diseases such as idiopathic intracranial hypertension (IIH). Importantly, to date, only one astronaut has reached the severity at which the risk to vision required consideration of intervention on the International Space Station (Brunstetter et al., 2024) and no astronaut has been required to be repatriated to Earth, based on their SANS status. As more astronauts become exposed to LDSF, the prevalence of SANS is expected to increase with longer durations of space flight. There may be the potential that some cases could progress to require intervention to avoid sight loss. In addition, little is known about the potential long‐term risks to astronaut ocular health, as the structural changes of chorioretinal folds, globe flattening, reduction in the length of the eye (axial length) and change to refractive error have not resolved in follow‐up, some documented over a decade following their mission (Roberts et al., 2019).

The aim of this narrative review is to highlight the potential for translating recent terrestrial medical knowledge to the main pathophysiological neuro‐ophthalmic changes associated with LDSF. The controversies and challenges of defining and investigating SANS will be presented, as will the potential countermeasures that could be considered for SANS.

## 2 IS SANS AN ORBITAL OR INTRACRANIAL DISORDER?

There has been debate over whether SANS is strictly an ocular or more broadly a neuro‐ophthalmic disorder with intracranial changes mediating the clinical signs seen in the eye.

### 2.1 Evidence that SANS is an ocular disorder

The first observations of ocular changes in astronauts during LDSF were published in 2011, and to date, two‐thirds of LDSF crew members have had one or more documented features of SANS (Macias et al., 2020; Mader et al., 2011). In a seminal report, a post‐flight questionnaire was administered to 300 flyers, where 29% of short (space shuttle) and 60% of LDSF astronauts described detrimental changes in their near and distance vision. This was followed by a case series of the seven LDSF astronauts who had undergone complete eye examinations, including refraction, fundus photography, OCT retinal imaging, brain MRI and lumbar punctures (LP). These findings were reported in part or completed before and after a 6‐month LDSF mission (Mader et al., 2011). Of the seven LDSF astronauts, six had retinal nerve fibre thickening on OCT imaging, with five having clinically apparent optic disc oedema on fundus imaging. Five had documented choroidal folds and five had globe flattening on MRI. The expansion of the choroid documented by OCT imaging (Pardon et al., 2022) was likely due to changes in venous drainage caused by microgravity. The choroid is the major vascular supply for the outer third of the retina, including the photoreceptors and retinal pigment epilethium (RPE), and while these choroidal folds and thickening have been reported, there does not currently appear to be a correlation between the extent of expansion and the development of optic nerve oedema. Small RPE detachments have also been reported in LDSF crew members, likely secondary to the choroidal changes termed pachychoroid.

Six astronauts experienced decreased near vision and had a hyperopic shift of between +0.50 and +1.75 dioptres in one or both eyes, likely due to the anatomical changes in the choroid, moving the position of the retina forwards. While cotton wool spots were documented in three astronauts, there has now been debate as to whether these should be part of the SANS clinical syndrome. Cotton wool spots are focal areas of ischaemia and these can be found in the periphery of the retina (as distributed in astronauts) or close to the optic disc, which may be related to ischaemia of the short posterior ciliary arteries or secondary to optic disc swelling.

Based on a case where choroidal expansion was documented over 30 days post‐mission and the asymmetric optic disc swelling persisted for some 180 days, some have considered that SANS is secondary to an orbital compartmentation syndrome, whereby fluid is retained in the peri‐optic subarachnoid space (Mader et al., 2017). This had previously been postulated in a case series of three patients with asymmetric papilledema secondary to IIH using computed cisternography with a contrast agent where a lack of contrast‐loaded cerebrospinal fluid (CSF) in the subarachnoid space of the less swollen optic nerve was observed despite the contrast being present in the intracranial subarachnoid space (Killer et al., 2007). However, some could consider that the changes in the choroid would have caused vascular remodelling due to venous overload, and therefore this may be a reason why choroidal folds do not resolve. At the present time, choroidal changes have not been demonstrated in bed rest studies, probably because while the 6‐degree head‐down tilt bed rest alters the primary gravity vector while lying supine, it cannot replicate weightlessness (Hargens and Vico, 2016).

### 2.2 Evidence that SANS is an intracranial pressure disorder

When exposed to μ‐gravity, there is a loss of the hydrostatic pressure gradient between the head and the feet. Astronauts typically experience facial swelling as the fluid shifts headward from the lower limbs. There is an apparent reduction in plasma volume as fluid shifts from the extravascular to the extracellular compartment and a lack of the normal homeostasis expected terrestrially (Drummer et al., 2000). Intracranial changes have been imaged in post‐flight MR studies. These have shown radiological signs of narrowing of the central sulcus and in CSF spaces at the vertex with an apparent upwards shift of the brain (Roberts et al., 2017). Also documented is an increase in the size of the lateral ventricles, loss of height and convexity of the pituitary gland and globe flattening in the presence of optic disc oedema. These findings are similar to conditions of raised intracranial pressure (ICP) commonly seen on Earth (Kramer et al., 2020; Sassani et al, 2025. In the follow‐up MR imaging study, an increase in the CSF volume and an increase in the cerebral aqueduct CSF flow velocity suggested a loss of brain compliance (Kramer et al., 2020).

Given the mild optic nerve oedema observed in SANS and our understanding of the non‐linear relationship of the intracranial volume and ICP (Mollan et al., 2023), it is likely that healthy astronauts have high compensatory reserves for dealing with increases in cerebral volume by reducing cerebral blood volume or displacing cerebrospinal fluid. It is therefore plausible that ICP could be raised above normal for that individual during LDSF, but only mildly. Lumbar punctures performed on four of the originally reported six astronauts found an opening pressure that ranged between 21 and 28.5 cmH_2_O measured between 12 and 60 days post‐mission (Mader et al., 2011). It is commonly accepted that the lumbar puncture opening pressure (LPOP) measurement is abnormal if it exceeds 25 cmCSF in adults (Mollan et al., 2018). Evidence here is provided by a large population study of older adults where the 95% reference interval for LPOP was found to be between 82 and 242 mmCSF and repeated LPs demonstrated that LPOP can vary considerably amongst individuals (Witsberger et al., 2022). To date, there have been no astronauts recruited to a pre‐ and post‐mission LP study to evaluate change in LPOP, which could aid in understanding of the raised ICP hypothesis. One challenge is that the LPOP should be measured at the time the person has active optic disc oedema and this would not be feasible on the International Space Station (ISS) for medical safety and the requirement for gravity to facilitate a measurement (Barr, 2014). Telemetric intracranial pressure monitors, like those that have been used in IIH (Mitchell et al., 2022), require a burr hole in the skull, which may be deemed too invasive to be deployed on mission for evaluation of SANS. It would be expected that the ICP should rapidly return to normal when astronauts return to Earth's gravity and hence why the four astronauts in whom an LP was performed post‐flight were found to be within the expected normal range.

Further evidence in the consideration that SANS is a CSF disorder comes from changes observed in the internal jugular veins (IJV), which are the major vessels for cranial blood outflow. The IJV play an important role on Earth, where it is the primary vein that carries the venous outflow while a person is supine. On standing, the IJV flow is reduced and CSF is redistributed to the spinal compartment; subsequently, the cerebral blood volume increases with an increase in ICP, thus preventing an acute substantial fall in ICP during the transition from supine to the upright position. Long‐duration space flight, between 4.0 and 5.5 months, has been shown to increase IJV volume (Arbeille et al., 2015); also, flow within the IJV has been shown to become static in crew members, with one having evidence of thrombosis and occlusion (Auñón‐Chancellor et al., 2020; Marshall‐Goebel et al., 2019). This reduction in venous outflow has been shown to increase central venous pressure and, in turn, cause intracranial venous hypertension. Intracranial venous hypertension causes CSF to be reabsorbed at higher ICP thresholds, thereby leading to raised ICP (Marshall‐Goebel et al., 2019).

Normal human physiology maintains a pressure gradient across the lamina cribrosa, which forms a border between the intraocular space and the retrobulbar space. The intraocular space has a higher pressure, and the retrobulbar cerebrospinal fluid space has a lower pressure, with the average pressure difference of 4 mmHg. Where there is either substantially reduced intraocular pressure (such as in hypotony) or elevated ICP, optic nerve head oedema can occur. Some have therefore cited that a reduced trans‐lamina cribrosa pressure gradient could be the source of the optic disc swelling in SANS. Investigators recruited healthy volunteers to measure lumbar puncture opening pressure (LPOP) and intraocular pressure in the supine and sitting position and a 9° head‐down tilt (HDT) to calculate the trans‐lamina cribrosa pressure difference and estimate this difference over a 24‐h period. They found that if a person does not have the predicted period of a differential in their ICP, due to being upright where the ICP is low and lying down where the ICP is higher, trans‐lamina cribrosa pressure difference could decrease due to the average ICP being higher than normal (Eklund et al., 2016). This could potentially cause disc oedema, globe flattening and choroidal folds.

## 3 POTENTIAL RISK FACTORS FOR RAISED INTRACRANIAL PRESSURE

There are a number of risk factors in space flight that could potentially cause raised ICP. These include resistive exercise, disturbances in circadian rhythm, chronic hypercapnia and alterations in the 1‐carbon metabolic pathway, the latter of which will be discussed in the context of SANS developing due to an apparent nutritional optic neuropathy.

### 3.1 Does resistive exercise cause raised intracranial pressure?

In space, resistive exercise is required to avoid musculoskeletal atrophy (Bosutti et al., 2025), and astronauts on the ISS typically spend 1.5 h a day, 6 days a week, using an advanced resistive exercise device for weightlifting. This exercise is intensive and may precipitate the Valsalva manoeuvre, which is known to increase arterial blood pressure, middle cerebral artery blood velocity and prefrontal cortex haemodynamics, which potentially could lead to raised ICP. Lawley et al. (2017) observed eight subjects with the ability to measure ICP through an Ommaya reservoir and noted central venous pressure and ICP increases during the contraction phase of leg press exercise if combined with a Valsalva manoeuvre; however, the ICP was noted to fall in the relaxation phase. This was observed in the supine position, in acute simulated μ‐gravity induced by parabolic flight and in prolonged simulated μ‐gravity with HDT bed rest. There is further supporting evidence that there is a transient ICP rise from a study utilising telemetric ICP monitors in women living with active IIH. When the Valsalva manoeuvre was performed, ICP temporarily increased for an average of 16 s and returned to baseline with no sustained impact on the ICP (Mitchell et al., 2022; Yiangou et al., 2023). It is therefore postulated that resistive exercise is unlikely to contribute to a raised ICP syndrome.

### 3.2 Relationship between circadian rhythm and intracranial pressure

Circadian rhythms are responsible for regulating various physiological processes that occur on an approximate 24‐h cycle; and these include sleep–wake patterns, hormone release and body temperature fluctuations. One challenge for astronauts is maintaining a regular sleep pattern and sleep duration. On the ISS, disturbances of light–dark cycles occur with 16 sunrises and sunsets in a 24‐h period. Specialised lighting is deployed on the ISS to mitigate this impact on circadian rhythm. Circadian rhythm is mediated by the light–dark cycle driven by the suprachiasmatic nucleus in the hypothalamus. It has been demonstrated that the choroid plexus, which produces the majority of CSF, is an integral component of the circadian system with the choroid plexus being a peripheral clock providing feedback to the suprachiasmatic nucleus (Myung et al., 2018).

Intracranial pressure is raised in patients with CSF disorders during sleep as compared to periods of wakefulness lying supine (Mitchell et al., 2022; Stephensen et al., 2006). It was hypothesised that raised ICP could be in part related to a change in posture, moving from supine to the prolonged prone position, associated with sleep (Andresen et al., 2015). However, a detailed study suggested that this may not be the case. Here, ICP was monitored continuously in intensive care patients with an external ventricular drain and contrasted to an in vivo experimentation with telemetric monitoring in experimental rodents. They found that the ICP increased in the dark phase in both humans and rats, most likely due to increased CSF secretion as demonstrated in the rodent model, and this was independent of posture and diurnal/nocturnal activity preferences (Steffensen et al., 2023). Validation of the effect of the dark cycle and relationship to CSF secretion would be of significant interest.

### 3.3 Is mild hypercapnia associated with raised intracranial pressure?

Ambient CO_2_ levels on the ISS have been reported to be mildly raised as compared to sea level on Earth and in the past have often risen above normal workplace recommendations (NASA, 2017). There is evidence that ISS astronauts have raised end‐tidal $P_{CO_{2}}$ levels (Hughson et al., 2016). Hypercapnia is known to cause cerebral vasodilatation, increasing cerebral blood flow and potentially increasing ICP (Brian J. E., 1998). During periods of raised $P_{CO_{2}}$ on the ISS, astronauts have reported increased headaches, but without an increase in clinically detectable SANS signs (Law et al., 2014). A 30‐day strict 6° HDT bed rest study exposed subjects to a mild hypercapnic environment to simulate the environment on the ISS. They found no increase in their end‐tidal $P_{CO_{2}}$ volume or level of $P_{CO_{2}}$ in arterial blood gas samples (Laurie et al., 2020). Another evaluation in strict HDT bed rest investigated whether mildly elevated CO_2_ levels influenced sleep and core body temperature and whether these were associated with the development of optic disc oedema. While sleep duration and temperature were not altered at 30 days of HDT bed rest, they found that shorter sleep duration, greater wake after sleep onset and lower core temperature amplitude present throughout the study increased the odds of developing optic disc oedema (Christian et al., 2024).

While modification of $P_{CO_{2}}$ levels on the ISS has subsequently occurred over the years, there has been no decline in the prevalence or incidence of SANS, which may suggest $P_{CO_{2}}$ is not a contributing factor. However, despite these data, the recently reported case of an astronaut with escalating SANS was noted to have a directed reduction in the mean $P_{CO_{2}}$. This was postulated to have contributed to the reduction in SANS findings on returning to Earth (Brunstetter et al., 2024).

## 4 IS SANS INFLUENCED BY THE ASTRONAUT'S DIET?

Nutrition continues to be a critical part of space medicine by providing vital energy and nutrients matched to the needs of the individual in this unique environment. While a comprehensive discussion is beyond the scope of this review, a few areas need to be highlighted in relation to the development of SANS. Initially, there was a concern with regard to the high salt content of an astronaut's diet. Not only is a high salt intake linked to increased risk of stroke and total cardiovascular disease (Strazzullo et al., 2009), but it can also contribute to increased bone resorption as demonstrated in bed rest studies of immobilised participants (Frings‐Meuthen et al., 2011). A comparison was made to IIH patients, where many clinicians have historically recommended a low salt diet for people living with IIH, to aid weight loss and induce disease remission, thereby reducing ICP. However, there is a lack of evidence for the use of a low salt diet for weight management, or indeed for lowering ICP (Abbott et al., 2023).

Neurological health is dependent on B vitamins (Gröber et al., 2013). Specifically, optic nerve function requires several key nutrients for normal mitochondrial metabolism, such as B_1_ (thiamine), B_6_ (pyridoxine), B_9_ (folate) and B_12_ (cobalamin). Evidence exists that the 1‐carbon metabolic pathway, which is folate‐ and vitamin B_12_‐dependent and involves homocysteine recycling and trans‐sulfuration, could be implicated as a risk factor for the development of SANS. This pathway has also been implicated in other ocular diseases such as Leber's hereditary optic neuropathy (Zibold et al., 2022) and nutritional optic neuropathy (Reynolds et al., 2022). In a study evaluating the nutritional and biochemical status of serum homocysteine, cystathionine, 2‐methylcitric acid and methylmalonic acid concentrations were found to be between 25% and 45% higher in those astronauts with SANS features than in those without them. The study also found lower serum folate in those astronauts with SANS. These differences were observed before, during and after flight (Zwart et al., 2016). Nutritional deficiencies were deemed unlikely to be the cause, as astronauts’ diet are pre‐planned for missions on the ISS. The diet provides folate, vitamin B_6_ and vitamin B_12_ in excess of the Institute of Medicine's recommended daily allowance (NASA, 2021). It therefore seems unlikely that SANS is mediated by diet alone.

### 4.1 Is SANS caused by an apparent nutritional optic neuropathy?

Nutritional optic neuropathy on Earth presents with loss of central vision and optic atrophy caused by mitochondrial dysfunction and impaired oxidative metabolism, which appears to be distinct from the clinical presentation of SANS (Sadun, 2002). However, a recent systematic review considered micronutrient deficiencies such as vitamin B_1_ and vitamin B_12_, which have noted optic disc swelling as a feature. They also found three cases where the LPOP was abnormally raised, which warrants further wider systematic evaluation of the nutritional status of those with conditions of CSF dysregulation, such as IIH (Reynolds et al., 2022). It could therefore be plausible that dysregulation in the 1‐carbon metabolism in LDSF astronauts could be a contributing factor to early disc swelling, as all astronauts have their eyes screened for SANS on orbit. This is in comparison to nutritional optic neuropathy on Earth, where the optic nerve swelling may be without symptoms at the early stage until the patient has symptoms of visual loss with optic atrophy.

Single‐nucleotide polymorphisms of one or more of a number of enzymes involved in folate‐ and vitamin B_12_‐dependent 1‐carbon metabolism are quite common and result in a moderately raised total homocysteine. The methylenetetrahydrofolate gene (*MTHFR*) encodes a critical enzyme, methylenetetrahydrofolate reductase. Without functional methylenetetrahydrofolate reductase, the multi‐step process of converting homocysteine to methionine does not occur (Frosst et al., 1995). Therefore, the amount of dietary folate needed to maintain normal folate status may be greater in individuals with the *MTHFR* C677T polymorphism (Zwart et al., 2012). *MTRR* 66 G and serine hydroxy methyltransferase gene (*SHMT*) 1420 C polymorphisms were found to be associated with the SANS ophthalmic changes following spaceflight in a case series of astronauts (Zwart et al., 2016). This study found that the *MTRR* 66 G allele was associated with an increased frequency of cotton wool spots and choroidal folds, whereas the *SHMT* 1420 C allele was associated with an increased prevalence of optic disc oedema (Zwart et al., 2016).

Folate deficiency may lead to hyperhomocysteinaemia even in the absence of vitamin B_12_ deficiency. Hyperhomocysteinaemia leads to the production of reactive oxygen species, causing endothelial dysfunction, vasoconstriction, platelet aggregation and in some a hypercoagulable state, leading to atherosclerosis and thrombus formation (Ray, 1998). There are a number of different ocular pathologies that may be potentiated by hyperhomocysteinaemia such as retinal vein occlusions, diabetic retinopathy and macular degeneration. There may be links to the report of stasis within the jugular vein and the thrombosis observed in one astronaut's internal jugular vein on screening (Auñón‐Chancellor et al., 2020; Marshall‐Goebel et al., 2019).

## 5 ANALOGOUS MODELS OF SANS

Analogous models have played an important part in understanding SANS. Mirroring knowledge from terrestrial diseases may be imperfect, but has provided insights into clinical assessment tools, potential therapeutic management and prognosis. Experimental studies utilising parabolic flight and HDT bed rest studies continue to be a valuable asset for progress (Table 1).

**TABLE 1:** Comparison of SANS features to the currently known terrestrial analogous models.

	Space flight‐associated neuroocular syndrome	Terrestrial diseases			Experimental analogues	
		Idiopathic intracranial hypertension	Idiopathic choroidal folds	Normal pressure hydrocephalus	Parabolic flight	Strict 6° head down tilt bed rest studies
Symptoms	Hypermetropic acuity shift; occasional mild headaches	Major symptoms include headaches and pulsatile tinnitus, and in some transient visual obscuration	May be asymptomatic or hypermetropic visual shift	Gait disturbance (20%) Urinary incontinence (15%) Cognitive decline Dementia (10%)	—	—
Ophthalmic findings	Optic disc oedema Posterior globe flattening Optic nerve sheath distension Posterior displacement of Bruch's membrane on OCT imaging Choroidal folds appear first, typically linear	Optic disc oedema Posterior globe flattening Optic nerve sheath distension Anterior displacement of Bruch's membrane on OCT imaging Retinal folds appear first, typically concentric	Prominent choroidal folds	None	Temporarily raised intraocular pressure (between 5–7 mmHg)	Optic disc oedema No choroidal thickening
MRI findings	Flattening of the globe Enlargement of the peri optic subarachnoid space Mild decrease in pituitary height Distended venous sinuses Ventricular expansion Upward shift of the brain	Intraocular protrusion and enhancement of optic nerve head Flattening of the globe Enlargement of peri peri‐optic subarachnoid space Increased vertical tortuosity of the optic nerve Decreased pituitary height Partially empty or empty sella Attenuation of the cerebral venous sinuses Slit‐like ventricles	Typically reported as normal for individual but may show Flattening of the globe Enlargement of peri optic subarachnoid space	Disproportionately enlarged subarachnoid space hydrocephalus (termed DESH) Ventriculomegaly Widening of the temporal horns of the lateral ventricle Acute callosal angle Upward bowing of the corpus callosum Dilated Sylvian fissure widening Transependymal oedema on MRI‐T2‐FLAIR can be demonstrated	—	Ventricular expansion Upwards brain shift
Lumbar puncture measurement	18–28.5 cmH_2_O post LDSF	>25 cmH_2_O Often >30cmH_2_O	Within normal range, i.e., <25 cmH_2_O	Normal LPOP, but CSF flow studies detail increased aqueduct CSF stroke volume and peak velocity	Acutely reduced compared to lying flat	Acutely increased proportional to the degree of head tilt

### 5.1 Terrestrial diseases as an analogue for SANS

Three main conditions have been considered to have SANS‐like features: IIH, idiopathic choroidal folds and normal pressure hydrocephalus. There are distinct differences in their clinical phenotype and examination findings (Table 1). Idiopathic intracranial hypertension is characterised by bilateral optic disc oedema secondary to raised intracranial pressure, with a risk of permanent visual loss and chronic headaches predominant. Adults who live with IIH are typically living with obesity and have been shown to have a profile of metabolic changes, endocrine dysfunction and cardiovascular risk profile distinct from that associated with obesity alone (Potter et al., 2024). While the risk profile and phenotype are expectantly different to SANS, many aspects can be translated, such as the non‐invasive technologies to monitor optic disc oedema (Mitchell et al., 2019). While the debate over whether ICP is central to SANS continues, the commonly used medications for IIH have been suggested for SANS, such as acetazolamide, topiramate and furosemide. All are likely to be contraindicated in space due to their direct action on the kidneys, secondary effects on bone metabolism, and for some, the documented disturbances of cognition (Mitchell et al., 2025).

### 5.2 Experimental studies as a method to study SANS

Head down tilt (HDT) bed rest studies use gravity to simulate headward fluid shifts that occur in space. Bed rest studies have been informing space medicine since the 1970s, but no SANS‐like features were observed in the initial studies. Use of a standard pillow under the head during HDT was sufficient to reintroduce the hydrostatic pressure gradient and reduce ICP by 4 mmHg (Lawley et al., 2017). In 2018, investigators used a strict protocol of 6‐degree HDT bed rest and raised CO_2_ levels where participants were not permitted to take breaks or use a pillow to prop up their head. During this study, 45% of participants developed SANS‐like optic disc oedema after 30 days (Laurie et al., 2019). Of key interest is that similar genetic polymorphisms in the 1‐carbon metabolism pathway that increased risks of SANS for astronauts were associated with the risk of developing optic disc oedema in an HDT bed rest study (Zwart et al., 2019).

## 6 COUNTERMEASURES TO ATTENUATE SANS

Despite the controversies surrounding the exact mechanism that causes SANS, strategies suggested to mitigate the effects of SANS involve either stimulating the effects of gravity or reducing ICP.

### 6.1 Evidence for countermeasures

Microgravity appears to be an essential component contributing to SANS. The ability to stimulate Earth's gravity in space could address SANS. Artificial gravity can be achieved by the linear acceleration or steady rotation of all or part of the spacecraft to stimulate gravity via the generation of centrifugal force. A rotating spacecraft would be extremely costly and challenging to engineer, but a human‐rated short‐radius centrifuge is a more practical solution in stimulating gravity via the generation of centrifugal force (Clément et al., 2015). A short‐radius centrifuge places the astronaut in the supine position with their head close to the rotational axis, with their feet pointing outwards. This device was trialled in 1998 during the Space Shuttle Neurolab mission (STS‐90) as the first and only in‐flight evaluation of artificial gravity on astronauts and suggested that centrifugal force of 0.5G applied longitudinally and 1G applied transversely were well tolerated by astronauts. Ocular changes and ICP were not investigated, but cardiovascular deconditioning appeared to have been reduced. The centrifuge was used for 20 min every other day during a 16‐day space mission, indicating its beneficial effect against μ‐gravity changes and potential use as a countermeasure for SANS (Clément et al., 2001).

Lower body negative pressure (LBNP) enables the recreation of hydrostatic effects from gravity by placing the legs and pelvic region in a semi‐airtight chamber with reduced pressure. This leads to a reduction in central venous pressure as blood and tissue fluids move from a relatively high‐pressure area to a relatively low‐pressure area, such as the lower body, to reduce the effects of SANS and raised ICP (Macias et al., 2015). Currently, Russian cosmonauts use a LBNP ‘Chibis’ suit as a countermeasure against post‐flight orthostatic intolerance on the ISS. Short duration LBNP and SANS findings have been evaluated on the ISS; however, they did not appear to impact the optic nerve head or choroidal changes. The investigators concluded that longer durations of a fluid shift reversal may be needed to impact the spaceflight‐induced ocular changes (Pardon et al., 2022). A key point is that no countermeasure should be considered in isolation. As an example, the use of LBNP is in part there to mitigate against the risks of space flight and lower the risk of thromboembolic events; however, it has been associated with activation of the coagulation cascade. A case of obstructive left internal jugular vein thrombosis was reported in an astronaut on an LDSF mission (Auñón‐Chancellor et al., 2020), and as intracranial venous outflow abnormalities have been documented in LDSF, it would be important to consider whether the use of LBNP in LDSF causes any additional risk of vascular thrombosis.

Given the evidence in both astronauts who develop SANS and in HDT bed rest participants who develop SANS‐like features that they have an underlying genetic predisposition to dysregulation in the 1‐carbon metabolism pathway, it would appear logical to test vitamin B complex supplementation (Zwart et al., 2012, 2016, 2019). The *B‐Complex: A Nutraceutical SANS Countermeasure* study is now recruiting astronauts that volunteer. A daily vitamin B complex supplement is taken for 6 months prior to flying, during the mission, and for 30 days after. The study investigators will take serum, test for genetic polymorphisms and compare these findings to the ocular examinations routinely performed on ISS (NASA, 2025). There was a recent case report of an astronaut on the ISS where the optic disc oedema had got to a level where it was deemed necessary to consider treatment; this led to two interventions: reducing CO_2_ exposure and a vitamin B complex supplement that folate, vitamin B6, B12 and riboflavin. On return to Earth the optic disc oedema was resolved (Brunstetter et al., 2024).

### 6.2 Potential novel countermeasure of interest for SANS

Glucagon‐like peptide‐1 (GLP‐1) receptor agonists are the most commonly prescribed medicine class in the United States and probably globally. They are licensed to treat type 2 diabetes mellitus and are also approved for weight management due to their effect on the hypothalamus to regulate weight and satiety. GLP‐1 receptors are also expressed in the choroid plexus and have been shown to reduce CSF secretion in a rodent hydrocephalus model (Botfield et al., 2017). This was subsequently translated to a phase 2 randomised controlled trial evaluating change in ICP measured by accurate telemetric ICP devices in women living with active IIH (Mitchell et al., 2023). Significant ICP reduction effect took place rapidly, seen within 2.5 h and throughout the pre‐planned endpoints of 24 h and 12 weeks, with a short‐acting GLP‐1 receptor agonist, exenatide. The magnitude of the ICP reduction appeared to be greater than conventionally used medicines to reduce ICP (Mitchell et al., 2025). During the study, nausea was the most commonly reported side effect; no changes were observed in laboratory values, vital signs or body mass index in the exenatide group (Mitchell et al., 2023). A secondary study found no negative impact on cognition (Grech et al., 2024). A study utilising GLP‐1 receptor agonist in HDT bed rest would be of high interest, as a GLP‐1 receptor agonist could be used to reduce CSF secretion, which may improve optic nerve oedema and brain fluid accumulation, irrespective of the underlying aetiology.

## 7 CHALLENGES ON THE HORIZON

Research directly on those who have SANS is limited due to the small and highly specific population capable of reaching space. Much of the research to date includes historical data with different methodologies and technology used to assess the clinical signs, and some of the data includes astronauts on repeated missions. Inclusion in hypothesis‐driven studies is on a voluntary basis, causing selection bias and limiting rapid progress. Given the limited space and chosen equipment in the ISS, measurement bias is also a risk. For example, certain tools, such as MRI, that have provided key information about post‐flight changes, would be helpful during flight, but currently would be impractical to deploy given the weight and space restrictions. Temporary biomarkers that could be critical to the pathogenesis of SANS could be overlooked due to pre‐planned scheduled tests on existing equipment. In addition, the μ‐gravity environment itself could influence the way certain measurements are interpreted, leading to potential errors or inconsistencies in the data collected if compared to terrestrial ‘normal’ values. With the advances in our ability to use real world data for machine learning and ability to generate synthetic data, it is likely more researchers will use artificial intelligence methods to predict, monitor and treat SANS in the future (Denniston et al., 2022; Herranz et al., 2024; Kamran et al., 2024).

There are currently mixed opinions about the prognosis of SANS. At present, the effects of optic nerve head oedema appear to be short‐lived and reversed upon return to Earth (Mader et al., 2011). However, the elasticity of the optic nerve sheath trabecular fibres or collagen structures may be permanently altered, and other non‐measurable structures might also be altered (Macias et al., 2020; Mader et al., 2020, 2017). There might also be a delayed onset of complications from the choroidal folds, such as neovascular age‐related macular degeneration. There is little literature for guidance regarding the long‐term effects of choroidal folds. Only through long‐term surveillance through the Longitudinal Study of Astronaut Health study will the risks associated with the astronauts' occupational exposures, as compared to the risks for a matched control population, be discovered (Institute of Medicine (US) Committee on the Longitudinal Study of Astronaut Health, 2004; NASA 2012).

Space‐flight associated neuroocular syndrome remains a ‘red’ risk for LDSF, but also for the mission to Mars and beyond (Bailey, 2025; Patel et al., 2020). The complexity of the interactions between the space ‘exposome’ and ‘integrome’ will require careful research to develop tolerable countermeasures to ensure safe passage (Bailey, 2025). While currently SANS is a top risk to be mitigated, the effects of chronic hypoxia on the eye in an extended LDSF to Mars may become a higher priority due to the known negative effects on function such as visual acuity and colour vision, and structural changes such as retinal neovascularisation, which can cause loss of vision (Wang et al., 2023).

## 8 CONCLUSION

SANS is a constellation of clinical features that presents heterogeneously amongst LDSF astronauts. This point alone suggests, what is not controversial, that the mechanism of SANS is most likely to be a multifactorial process. Probably, individual risk factors, such as the 1‐carbon metabolism pathway, coupled with the μ‐gravity environment where cephalic fluid shifts are unable to be dealt with physiologically, may cause the condition. Whether the ICP is pathologically raised continues to be debated. However, there is evidence suggesting that CSF is dysregulated, likely secondary to a lack of the normal postural changes that occur in 24 h in space; a reduction of venous outflow; and potentially the role of the choroid plexus in the light–dark cycle. There are confounding factors that may influence the development of SANS, such as radiation exposure, psychological stress and changes in the environment that could interact in ways not currently understood. These may impact the true relationship between μ‐gravity and the development of SANS.

Evidence‐based countermeasures, such as the application of lower body negative pressure, are emerging but are cumbersome on the astronaut's time. Further evidence is required regarding the relationship between nutritional supplementation and optic disc oedema. Bed rest studies remain a robust way of providing surrogate information that can be translated to space flight. A significant challenge of designing trials with analogue models for SANS includes whether to randomise participants to the countermeasure and placebo, where not all participants will develop features analogous to SANS, or providing the countermeasure only when SANS‐like features occur. This will, of course, mirror the debate space agencies and flight surgeons will have when planning to deploy suitable countermeasures: preventative medicine versus personalised medicine. This discussion will be informed by the acceptability of the countermeasure and further knowledge regarding the long‐term risks to astronaut health that SANS poses.

## AUTHOR CONTRIBUTIONS

Vincent Wing Sum Ng: literature review; first draft of the manuscript. Susan Patricia Mollan: concept, creation of the table, further literature review and final drafting of the manuscript. All authors have read and approved the final version of this manuscript and agree to be accountable for all aspects of the work in ensuring that questions related to the accuracy or integrity of any part of the work are appropriately investigated and resolved. All persons designated as authors qualify for authorship, and all those who qualify for authorship are listed.

## CONFLICT OF INTEREST

V.N. declares no conflicts of interest. S.P.M. reports grants or research income paid to her institution from the NIHR, UK Space Agency and IIH UK, and personal fees from Invex Therapeutics, Teva and Thea Pharmaceuticals and non‐financial support from Heidelberg Engineering, outside the submitted work.

## FUNDING INFORMATION

None.
